# Doxorubicin Induces Cytotoxicity through Upregulation of pERK–Dependent ATF3

**DOI:** 10.1371/journal.pone.0044990

**Published:** 2012-09-13

**Authors:** Eun-Jung Park, Hyuk-Kwon Kwon, Yong-Min Choi, Hyeon-Jun Shin, Sangdun Choi

**Affiliations:** Department of Molecular Science and Technology, Ajou University, Suwon, Korea; National Institutes of Health, United States of America

## Abstract

Although doxorubicin is commonly used in the treatment of many cancer types, its use in chemotherapy has been limited, largely because of its severe side effects, including cardiotoxicity and nephrotoxicity. In this study, we aimed to identify the mechanism of doxorubicin-induced cytotoxicity by using the human kidney proximal tubule cell line HK-2. Furthermore, we investigated the role of activating transcription factor 3 (ATF3) as a mediator of doxorubicin-induced cytotoxicity by using wild-type mouse embryonic fibroblasts (MEF) cells and ATF3 knockout (KO) cells. In HK-2 cells, doxorubicin decreased cell viability in a dose-dependent manner and induced an increase in cells in the sub G1 and G2/M phases at all doses. Doxorubicin treatment showed the following dose-dependent effects: increase in the secretion of tumor necrosis factor alpha; decrease in the expression of phosphorylated protein kinase A and Bcl-2; and increase in the expression of phosphorylated signal transducer and activator of transcription 3, phosphorylated extracellular signal-regulated kinase (ERK), and ATF3. Based on these results, we suggest that doxorubicin induces cytotoxicity through an ERK-dependent pathway, and ATF3 plays a pivotal role as a transcriptional regulator in this process.

## Introduction

Since its discovery in 1971, doxorubicin, which intercalates with DNA, is one of the most widely used cancer chemotherapeutic agents. Although doxorubicin is commonly used in the treatment of a wide range of cancers, including hematological malignancies, different types of carcinomas, and soft-tissue sarcomas, its use in chemotherapy has been limited, largely because of its severe side effects, including cardiotoxicity and nephrotoxicity [1]–[3].

Reactive oxygen species generation and lipid peroxidation have been suggested to be responsible for doxorubicin-induced cardiotoxicity and nephrotoxicity [2], [4]–[7]. The formation of an iron-anthracycline complex that generates free radicals, which, in turn, causes various forms of oxidative damage to critical cellular components and to membrane lipids in the plasma membranes and mitochondria, has been associated with doxorubicin-induced renal damage [4], [8]–[13]. In addition, the activation of mitogen-activated protein kinase (MAPK) pathways is involved in the development of doxorubicin-induced cardiomyopathy. For example, the activation of p38 MAPK induces the apoptosis of cardiac cells, while the extracellular signal-regulated kinases (ERK) and Jun-N-terminal kinases (JNK) are known to inhibit apoptotic cell death 7,14–18. Pro-apoptotic proteins such as Fas, anti-apoptotic proteins such as Bcl-2, and the tumor suppressor protein p53 are also involved in doxorubicin-induced apoptosis [11], [16], [19]–[22].

Cisplatin, an anti-cancer drug that causes nephrotoxicity, has been previously shown to induce cytotoxicity via activating transcription factor 3 (ATF3) together with the MAPK pathway [23]. ATF3, a member of the ATF/cyclic adenosine monophosphate (cAMP)-responsive element-binding protein (ATF/CREB) family of transcription factors, is a stress-inducible transcriptional repressor. It is induced in response to cytotoxic stress stimuli and is closely related to the regulation of the MAPK cascade [24], [25]. However, it is unclear whether ATF3 plays a pro-apoptotic or anti-apoptotic role in doxorubicin-induced nephrotoxicity. In this study, we aimed to identify the cause of doxorubicin-induced cytotoxicity by using the human kidney proximal tubule cell line HK-2. Furthermore, we investigated the role of ATF3 as a mediator of doxorubicin-induced cytotoxicity by using wild-type and ATF3 knockout (KO) mouse embryonic fibroblast (MEF) cells.

## Materials and Methods

### Cell Culture

HK-2, a proximal tubular cell line derived from normal kidney, was purchased from the American Type Culture Collection (ATCC; Manassas, VA, USA) and the MEF and ATF3 KO mouse cell lines were previously established in our laboratory as described in Kim *et al* [26]. Cells were cultured in 10-mm plates by using the media recommended by the ATCC, antibiotics (1% penicillin/streptomycin), and 10% heat-inactivated fetal bovine serum in a 37°C humidified incubator containing 5% CO_2_.

**Table 1:** List of primer sequences used in this study.

Symbol	Sequence	
ATF3	F	5'-GAGGATTTTGCTAACCTGACGC-3'
	R	5'-CTACCTCGGCTTTTGTGATGG-3'
P53	F	5'-GAGGTTGGCTCTGACTGTACC-3'
	R	5'-TCCGTCCCAGTAGATTACCAC-3'
MDM2	F	5'-GAATCATCGGACTCAGGTACATC-3'
	R	5'-TCTGTCTCACTAATTGCTCTCCT-3'
CDK2	F	5'-GCTTTCTGCCATTCTCATCG-3'
	R	5'-GTCCCCAGAGTCCGAAAGAT-3'
CDK4	F	5'-ACGGGTGTAAGTGCCATCTG-3'
	R	5'-TGGTGTCGGTGCCTATGGGA-3'
IL-6	F	5'-ATGAACTCCTTCTCCACAAGCGC-3'
	R	5'-GAAGAGCCCTCAGGCTGGACTG-3'
GAPDH	F	5'-GGGGTGAGGCCGGTGCTGAGTAT-3'
	R	5'-TGGGGGTAGGAACACGGAAGG-3'

**Figure 1:**
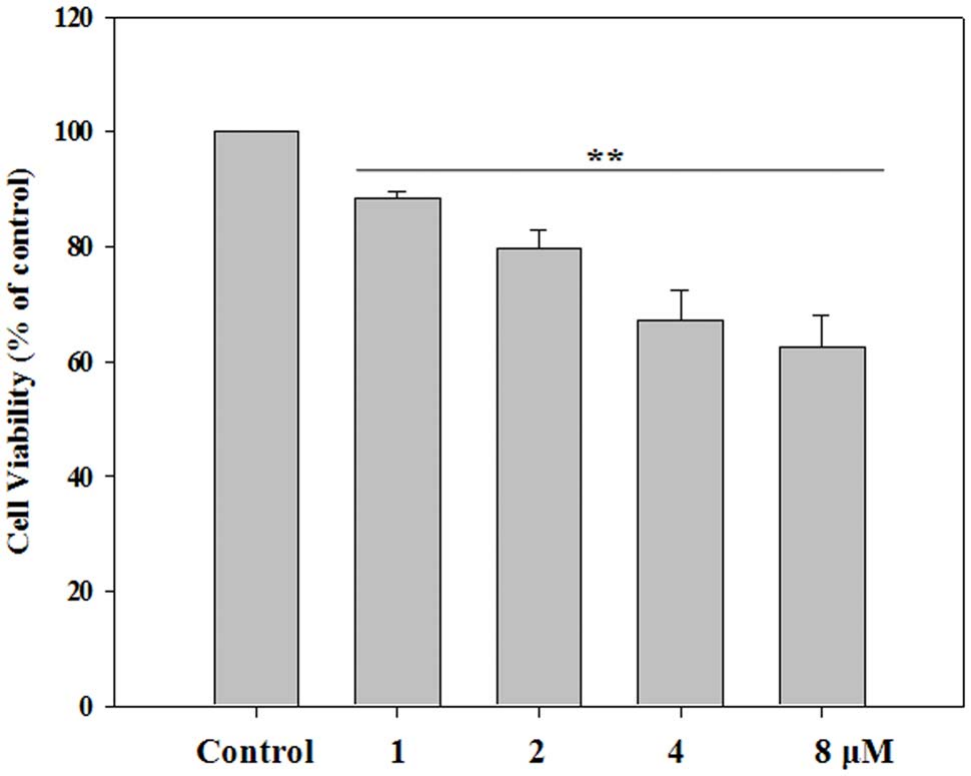
Proliferation of HK-2 cells. HK-2 cells were treated with 1, 2, 4, or 8 µM doxorubicin for 24 h. 3-(4,5-Dimethylthiazol-2-yl)-5-(3-carboxymethoxyphenyl)-2-(4-sulfophenyl)-2H-tetrazolium solution was added, and the UV absorbance of each treated group was calculated as a percentage of the control value. Experiments were independently performed 5 times. **P<0.01, *P<0.05.

### Cell Proliferation Assay

Cell proliferation was determined using a colorimetric 3-(4,5-dimethylthiazol-2-yl)-5-(3-carboxymethoxyphenyl)-2-(4-sulfophenyl)-2H-tetrazolium (MTS) solution (Cell Titer 96®; Promega, Madison, WI, USA). Cells (1×10^4^ cells/well) were seeded on 96-well plates, allowed to stabilize for 24 h, and then treated with 1, 2, 4, or 8 µM doxorubicin for 24 h. At the end of the exposure, the MTS solution (20 µL/well) was added, and the cells were incubated for 3 h at 37°C. The optical density of each well at 490 nm was then measured using a microplate spectrophotometer system (VersaMax; Molecular Devices, Sunnyvale, CA, USA).

**Figure 2:**
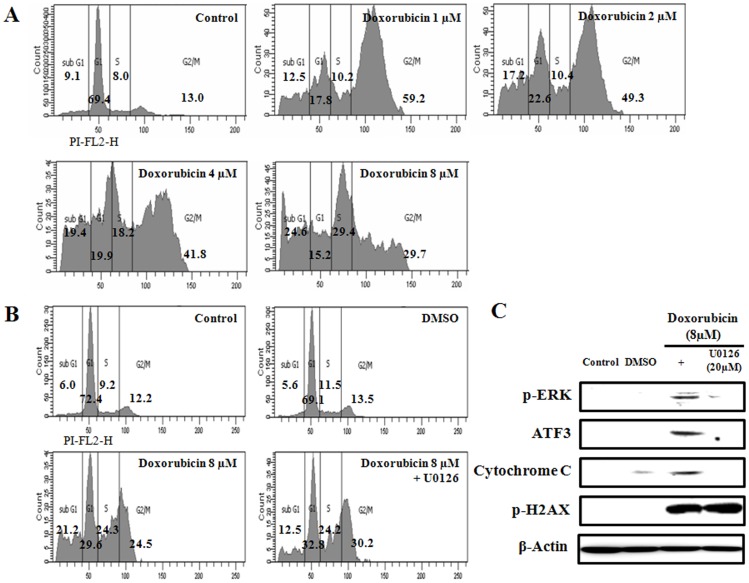
Cell cycle arrest of HK-2 cells. (A) HK-2 cells were treated with 1, 2, 4, or 8 µM doxorubicin. After 24 h of treatment, cells were harvested and stained with propidium iodide solution after ethanol fixing. (B) HK-2 cells were pretreated with the ERK inhibitor U0126 (25 µM) for 30 min, followed by treatment with 8 µM of doxorubicin for 24 h. Experiments were independently performed 3 times, and representative data are shown. (C) HK-2 cells were pretreated with ERK inhibitor (U0126, 25 µM) for 30 min. Subsequently, the cells were treated with 8 µM of doxorubicin for 24 h. Whole-cell extracts were analyzed for p-ERK, ATF3, cytochrome C and gamma-H2AX by Western blot.

### Nitric Oxide Analysis

Cells were plated at a density of 1×10^6^ cells/mL into 96-well plates with or without doxorubicin and incubated for 24 h. Supernatant (100 µL) from each well was transferred to new 96-well plates and was tested using a nitric oxide (NO) detection kit (iNtRON Biotech, Gyeonggi-do, Korea). Values were calculated by measuring the absorbance at 540 nm by using the microplate spectrophotometer system (VersaMax; Molecular Devices).

**Figure 3:**
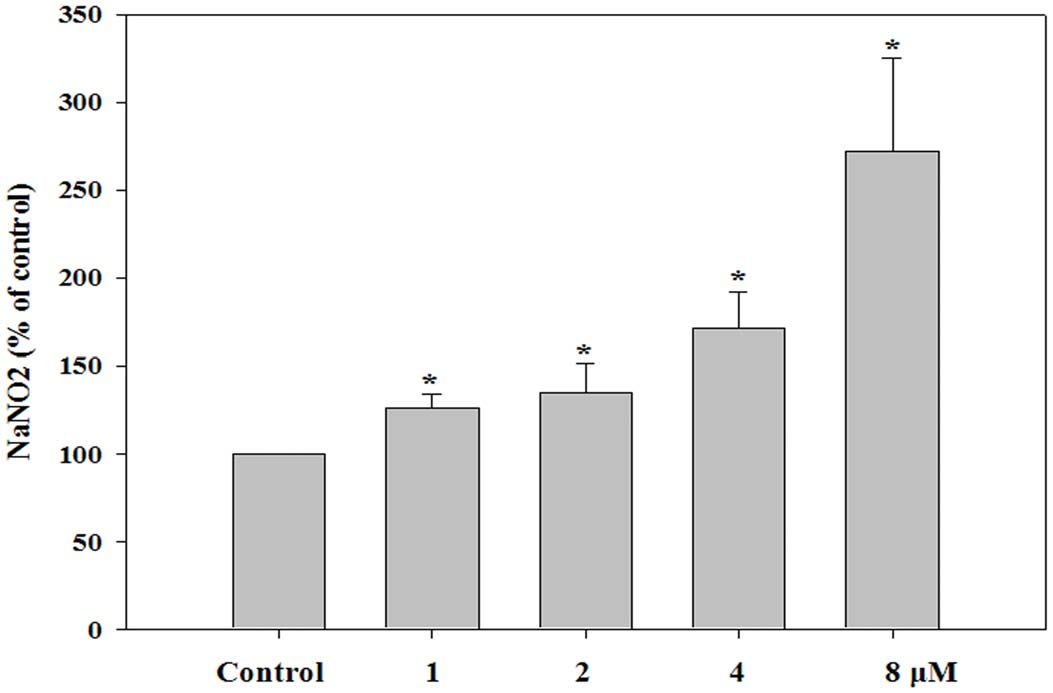
Nitric oxide secretion by HK-2 cells. HK-2 cells were treated with 1, 2, 4, or 8 µM of doxorubicin. After 24 h, the cell culture supernatant was reacted with an equal amount of Griess reagent. The amount of NO was calculated using a standard curve. Experiments were independently performed 5 times. **P<0.01, *P<0.05.

### Cell Cycle Analysis

Cells were stained using propidium iodide (500 µL) and RNase, which was purchased from Sigma-Aldrich (St. Louis, MO, USA). The cell cycle was analyzed by measuring the DNA content by using the FACSCalibur system and CellQuest software (BD Biosciences, Franklin Lakes, NJ, USA).

### Reverse-transcriptase Polymerase Chain Reaction

Total RNA was isolated by RNeasy mini kit (Qiagen, Valencia, CA, USA), and the total RNA concentration was detected using a Micro UV-Vis fluorescence spectrophotometer (Malcom, Tokyo, Japan). Total RNA (1 µg) was converted to cDNA by using the cDNA Synthesis Master Mix (GenDEPOT; Barker, TX, USA). Polymerase chain reaction (PCR) was performed using the GoTaq Green Master Mix (Promega). Thermal cycling was carried out with an initial denaturation phase of 2 min at 95°C, followed by 30 cycles of 30 s at 95°C, 30 s at 55°C, and 1 min at 72°C, with a final extension cycle of 5 min at 72°C. Amplification was carried out in the Veriti 96-well Thermal Cycler (Applied Biosystems). The final products were resolved on a 1% agarose gel stained with ethidium bromide. Primers used in this study are shown in Table 1.

**Figure 4:**
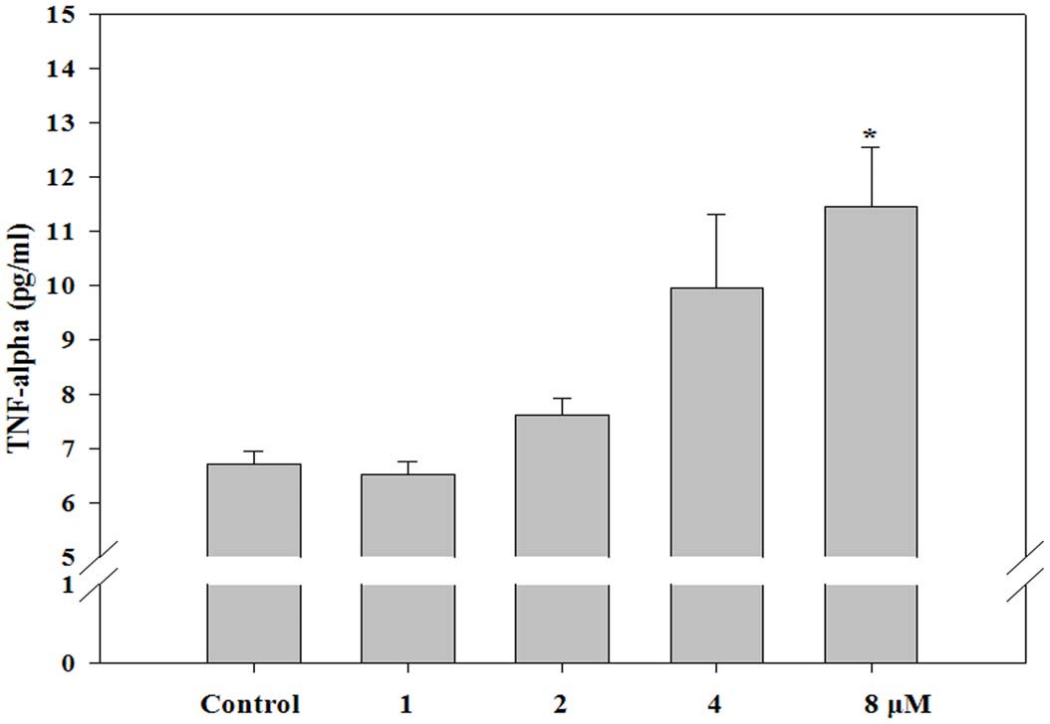
Tumor necrosis factor-alpha secretion by HK-2 cells. HK-2 cells were treated with 1, 2, 4, or 8 µM of doxorubicin. After 24 h, the concentration of tumor necrosis factor-alpha (TNF-α) in the cell culture supernatant was measured using an ELISA kit. The TNF-α concentration was calculated using a standard curve. Experiments were independently performed 3 times. **P<0.01, *P<0.05.

### Western Blot Analysis

Cell pellets were homogenized with a protein extraction solution (PRO-PREP™; iNtRON Biotechnology, Korea), and the lysates were centrifuged at 15,000×*g* for 10 min. The protein concentration was measured by the Bradford method (Bio-Rad Protein Assay, Bio-Rad Laboratories Inc., Hercules, CA, USA), and equal amounts of protein (40 µg) were separated on a 1% sodium dodecyl sulfate-polyacrylamide gel, and then transferred to a nitrocellulose membrane (Hybond ECL; Amersham Pharmacia Biotech Inc., Piscataway, NJ, USA). Blots were blocked for 2 h at room temperature with 5% (w/v) non-fat dried milk in Tris-buffered saline (10 mM Tris, pH 8.0, and 150 mM NaCl) solution containing 0.05% Tween-20. The membranes were immunoblotted with the following specific primary antibodies (1∶1000 dilution): rabbit polyclonal antibodies for p-STAT3(S), p-ERK, p-p38, p-JNK, p-p53, p-STAT3(Y) (Cell Signaling Technology, Inc. Beverly, MA, USA), ATF3, γ-H2AX (phosphorylated H2AX) (Santa Cruz Biotechnology Inc., Santa Cruz, CA, USA), p-CREB (Upstate Chemicon, Temecula, CA, USA), and BID (Chemicon International, Inc. Temecula, CA, USA); rabbit monoclonal antibodies for BAD, p-PKA (Upstate Chemicon), Apaf-1, and BAX (Millipore, Billerica, MA, USA); mouse monoclonal antibodies for Bcl-2 (Upstate Chemicon), cytochrome C (Upstate Chemicon), MDM2, and β-actin (Santa Cruz Biotechnology Inc.). The blots were then incubated with the corresponding conjugated anti-mouse, anti-rabbit, or anti-goat immunoglobulin G-horseradish peroxidase (1∶2,000 dilution; Santa Cruz Biotechnology Inc.). Immunoreactive proteins were detected with the ECL western blotting detection system.

**Figure 5:**
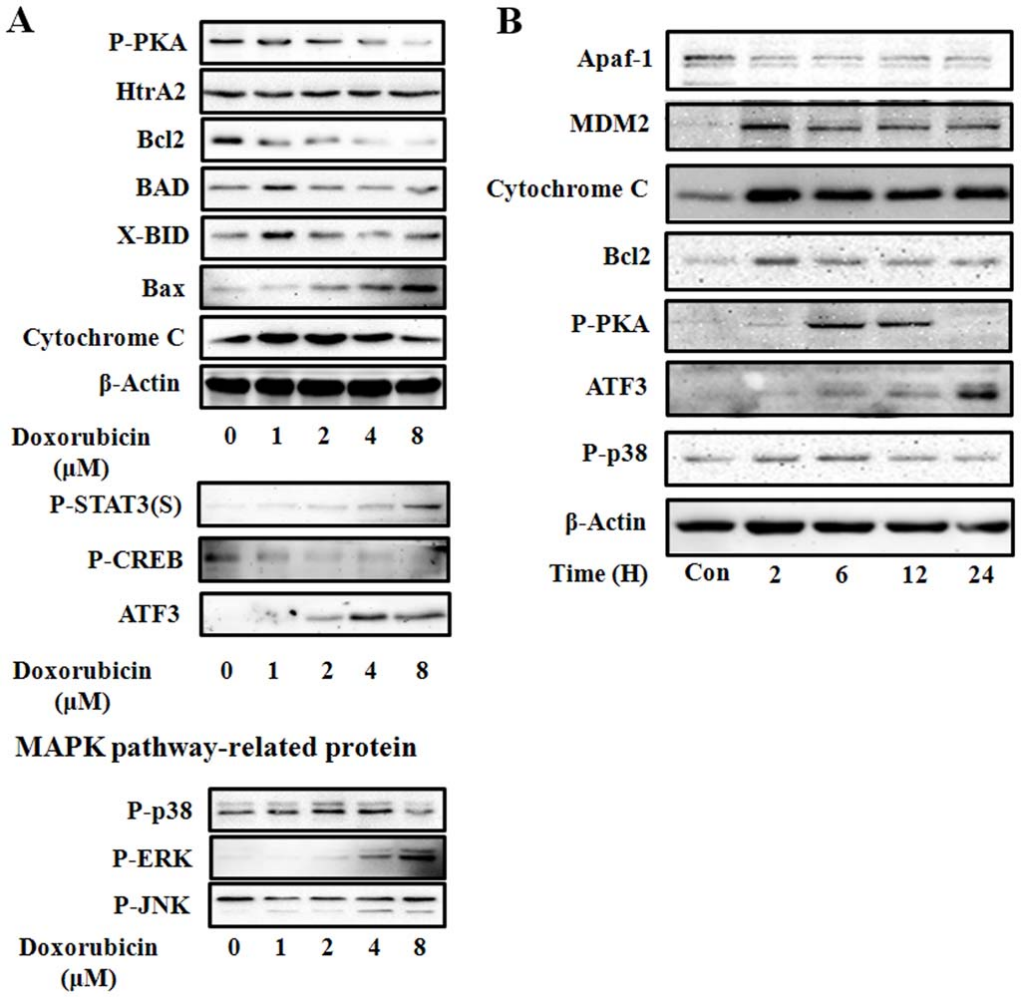
Protein expression in HK-2 cells. HK-2 cells were treated with 1, 2, 4, or 8 µM of doxorubicin. After 24 h of treatment, live cells were harvested, and the cell lysate was reacted with the indicated antibodies (A and B). Experiments were independently performed 3 times, and representative data are shown.

### Cytokine Analysis

The concentration of each cytokine in the supernatant of the culture media and serum was determined using commercially available enzyme-linked immunosorbent assay (ELISA) kits (eBioscience). First, each well in the microplate was coated with 100 µL of capture antibody and incubated overnight at 4°C. After washing and blocking with assay diluent and bronchoalveolar lavage fluid, the serum or standard antibody was added to the individual wells. The plates were then maintained at room temperature for 2 h. The plates were washed, and then, biotin-conjugated-detecting antibody was added to each well, and the plates were incubated at room temperature for 1 h. After incubation, the plates were washed again and further incubated with avidin-horseradish peroxidase for 30 min before detection with 3,3′,5,5′-tetramethylbenzidine solution. Finally, the reactions were stopped by adding 1 M H_3_PO_4_, and the absorbance at 450 nm was measured with an ELISA reader (Molecular Devices). The amount of cytokine was calculated from the linear portion of the generated standard curve [27], [28].

**Figure 6:**
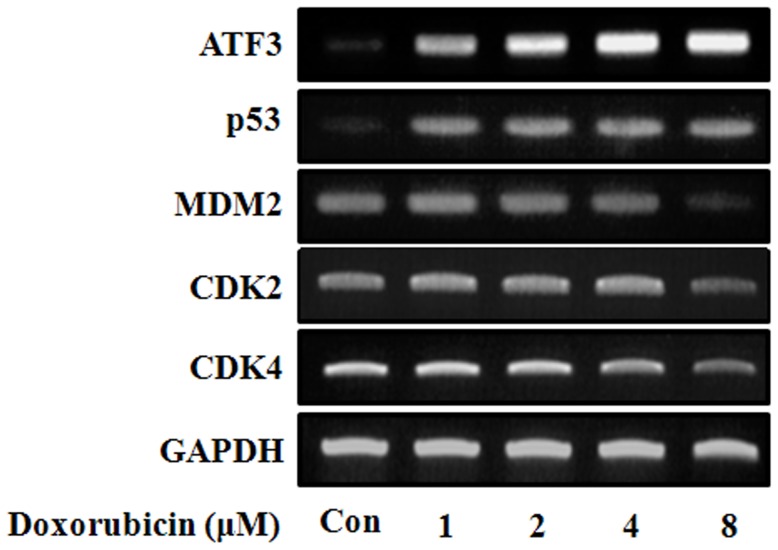
mRNA expression in HK-2 cells. HK-2 cells were treated with 1, 2, 4, or 8 µM of doxorubicin. After 24 h of treatment, live cells were harvested, and cell lysates were subjected to reverse-transcriptase polymerase chain reaction with the specific primers shown in Table 1. Experiments were independently performed 3 times, and representative data are shown.

### ATF3 Silencing using siRNA

siRNA (5′-CTGTGAGATAAGCGGGACTCAG-3′) for ATF3 and scrambled siRNA were purchased from Genolution Pharmaceuticals (Seoul, Korea). G-Fectin (Genolution Pharmaceuticals) was used as the siRNA delivery reagent. HK-2 cells were seeded at a density of 1×10^6^ cells in 6-cm dishes containing 2 mL of Keratinocyte-SFM (Invitrogen, Carlsbad, CA, USA) overnight. PBS (200 mL) containing 10 nM of siRNA and 4 µL of G-Fectin reagent was incubated at room temperature for 10 min and added to each well. After the transfection with siRNA for 48 h, the medium was replaced with normal medium and used for subsequent experiments.

**Figure 7:**
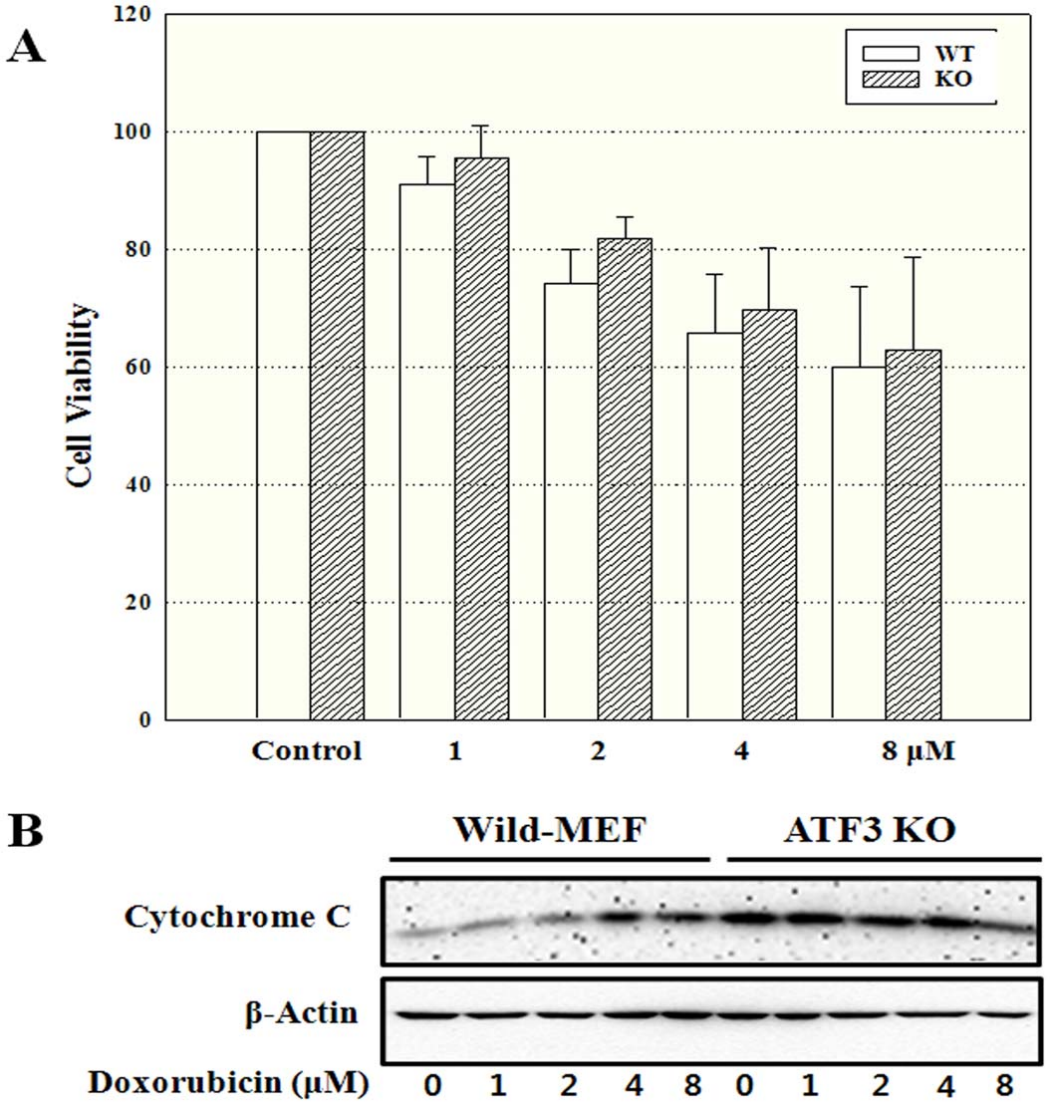
Cell proliferation in wild-type mouse embryonic fibroblasts and activating transcription factor 3 knockout cells. (A) Wild-type mouse embryonic fibroblasts (MEFs) and activating transcription factor 3 knockout (ATF3 KO) cells were treated with 1, 2, 4, or 8 µM doxorubicin for 24 h. The measured UV absorbance was expressed as a percentage of the control value. Experiments were independently performed 5 times. P<0.05. (B) Wild-type MEFs and ATF3 KO cells were treated with 1, 2, 4, or 8 µM of doxorubicin, for 24 h. Cell lysates from live cells were reacted with the indicated antibodies. Experiments were independently performed 3 times.

### Statistical Analysis

Statistical analyses were performed using the Statistical Package for the Social Sciences v. 12.0 (SPSS; http://www.spss.com). Each datum represents the mean ± S.E.M. of the different experiments under the same conditions. Statistical significance was calculated between each treated group and the control by the independent *t*-test. P values less than 0.05 were considered statistically significant.

**Figure 8:**
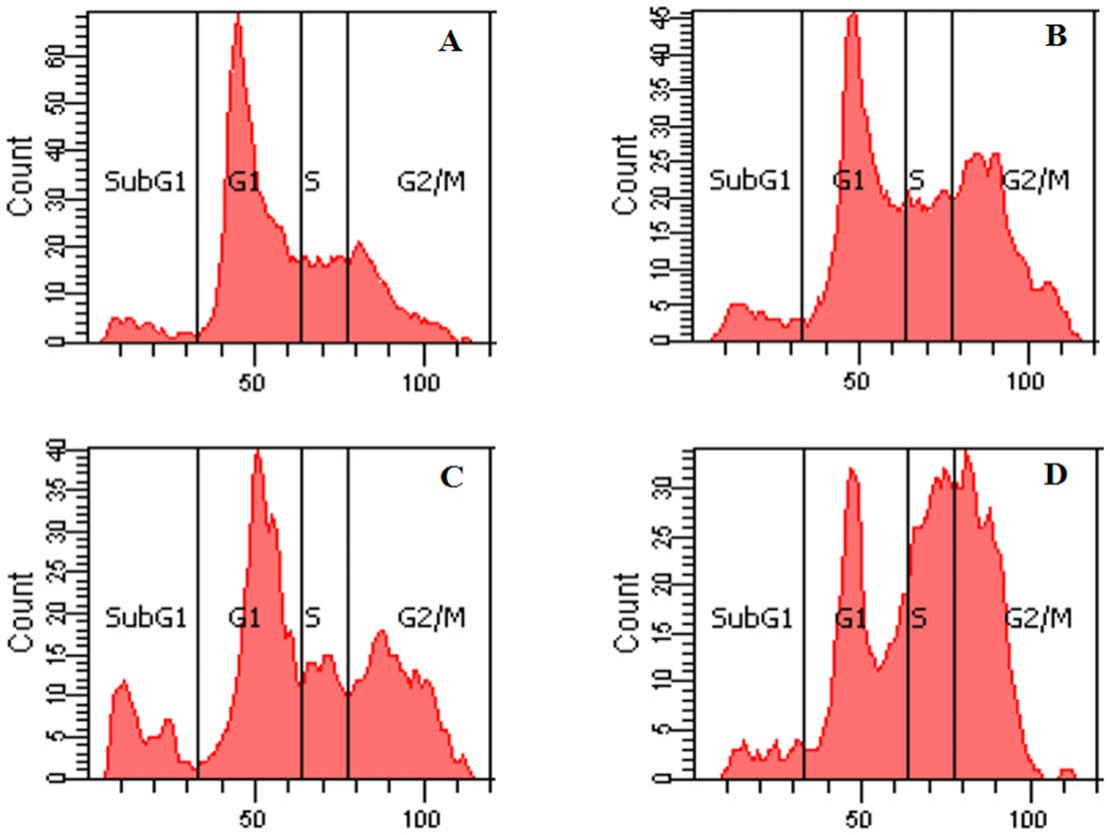
Cell cycle arrest of wild-type MEFs and ATF3 KO cells. Wild-type MEF and ATF3 KO cells were treated with 8 µM doxorubicin for 24 h (A, wild-type control; B, wild-type treated with doxorubicin; C, ATF3 KO control; D, ATF3 KO treated with doxorubicin). Experiments were independently performed 5 times, and representative data are shown.

## Results

### Cell Proliferation and Cell Cycle in HK-2 Cells

To investigate the effect of doxorubicin on HK-2 cells, we treated HK-2 cells with 1, 2, 4, or 8 µM doxorubicin for 24 h. Cell proliferation decreased in a dose-dependent manner upon doxorubicin treatment to 62.5%±5.3% of control at the 8 µM dose (Figure 1). Further, doxorubicin treatment at all doses tested caused an increase in the number of cells in the sub G1 (cell death) and G2/M phases (Figure 2A). We utilized a pharmacological ERK inhibitor, U0126 to evaluate the cell cycle after doxorubicin treatment. Cell proliferation was recovered on treatment with this inhibitor, indicating that ERK signaling relates to cell death caused by doxorubicin (Figure 2B). Furthermore, it is also evident in Figure 2C that the inhibition of ERK signaling downregulates the expression of ATF3 and cytochrome C.

**Figure 9:**
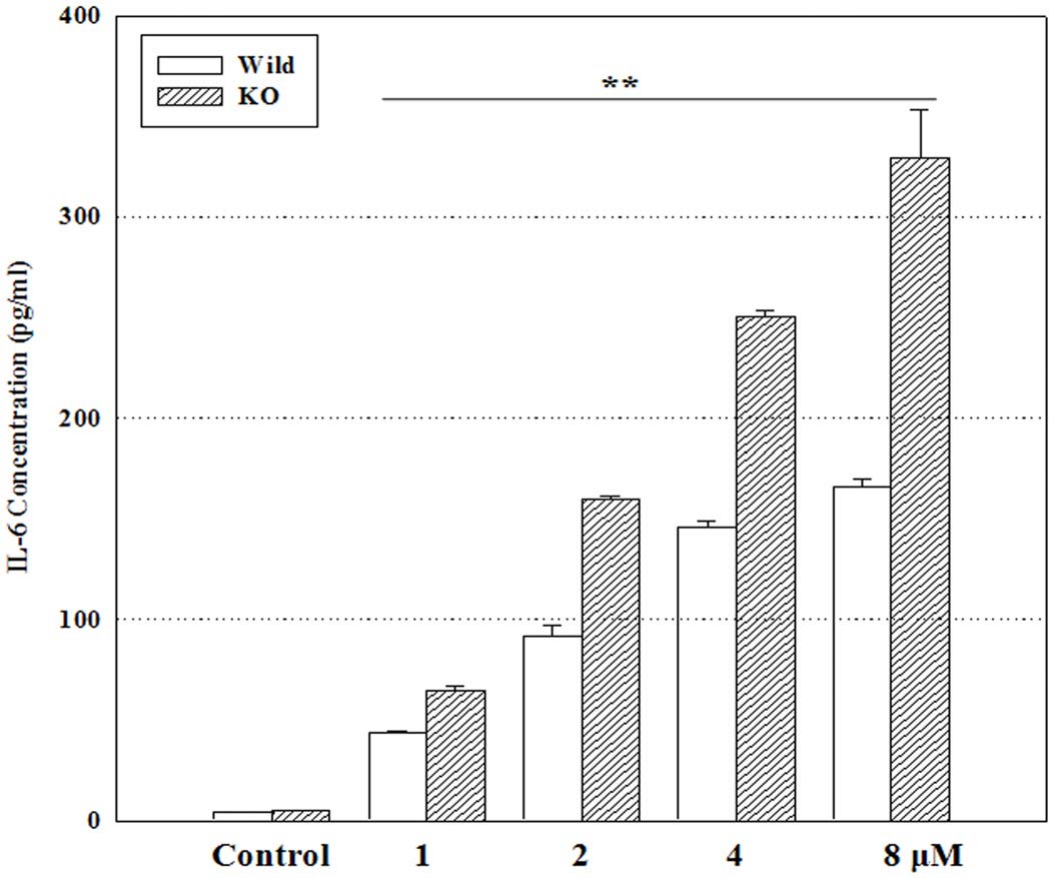
Interleukin-6 concentration in wild-type MEFs and ATF3 KO cells. Wild-type MEFs and ATF3 KO cells were treated with 1, 2, 4, or 8 µM doxorubicin, for 24 h. The concentration of interleukin-6 (IL-6) in the cell culture supernatant was measured using an enzyme-linked immunosorbent assay kit. The IL-6 concentration was calculated using a standard curve. Experiments were independently performed 5 times. **P<0.01, *P<0.05.

### Nitric Oxide Secretion in HK-2 Cells

Treatment of HK-2 cells with doxorubicin caused an increase in the concentration of NO in a dose-dependent manner, with values corresponding to 125.8%±7.9%, 135.0%±16.1%, 171.6%±20.6%, and 271.8%±53.7% of control treatment with 1, 2, 4, and 8 µM doxorubicin, respectively (Figure 3).

### Tumor Necrosis Factor-alpha Secretion in HK-2 Cells

As shown in Figure 4, the secretion of tumor necrosis factor-alpha (TNF-α) increased in a dose-dependent manner, with values of 6.5±0.2, 7.6±0.3, 9.9±1.4, and 11.5±1.1 pg/mL upon treatment with 1, 2, 4, and 8 µM doxorubicin, respectively, compared with 6.7±0.2 pg/mL in control cells.

**Figure 10:**
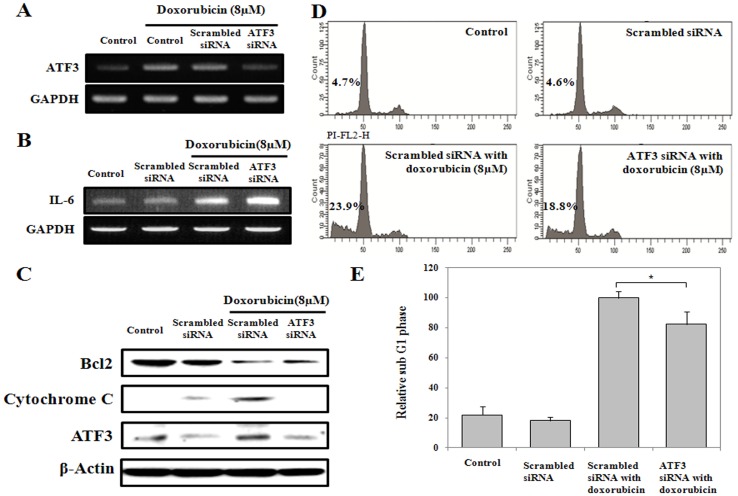
ATF3 silencing in HK-2 cells by using siRNA. HK-2 cells were transfected with either scrambled or ATF3 siRNA. (A) ATF3 mRNA levels were decreased in cells treated with ATF3 siRNA when compared with scrambled siRNA treated cells. (B) IL-6 mRNA levels were determined by RT-PCR. (C) Protein levels of Bcl2, cytochrome C and ATF3 were measured by Western blot. (D) The cell cycle was analyzed by measuring the DNA content by using the FACSCalibur system in HK-2 cells after treatment of ATF3 or scrambled siRNA for 48 h and treatment with or without doxorubicin (8 µM) for 24 h. (E) Histograms represent the relative mean ratios of the cells in sub G1 phase from 3 independent experiments. *P<0.05.

### Protein and mRNA Expression in HK-2 Cells

We investigated the effect of doxorubicin treatment on the expression of several proteins (Figure 5). We found that doxorubicin decreased the expression of phosphorylated protein kinase A (phospho-PKA) and Bcl-2 in a dose-dependent manner, while it increased the expression of phosphorylated signal transducer and activator of transcription 3 (phospho-STAT3), phospho-ERK, and ATF3 in a dose-dependent manner. Further, the expression of MDM2 and Bcl-2 decreased following a peak 2 h after treatment; expression of phospho-PKA and phospho-p38 peaked at 6 h after treatment. The expression of ATF3 and p53 mRNA also increased (Figure 6).

### Cell Proliferation and Protein Expression in Wild-type and ATF3 KO MEF Cells

To investigate the role of ATF3 in doxorubicin-induced cytotoxicity, we compared cell proliferation using wild-type and ATF3 KO MEFs (Figure 7A). Decreased cell proliferation was more pronounced in the ATF3 KO cells compared to the wild-type cells. The percentage of surviving cells relative to control was higher in ATF3 KO MEFs than in the wild-type MEFs 24 h after treatment with doxorubicin. Further, the expression of cytochrome C decreased in a dose-dependent manner in ATF3 KO cells, but increased in a dose-dependent manner in wild-type MEFs (Figure 7B).

### Cell Cycle in Wild-type and ATF3 KO MEFs

As shown in Figure 8, doxorubicin induced cell-cycle arrest at the S and G2/M phases in both wild-type and KO cells. However, distribution of cells in the sub G1 phase decreased significantly in ATF3 KO cells.

### Interleukin (IL)-6 Concentration in Wild-type and ATF3 KO MEFs

As shown in Figure 9, the concentration of IL-6 increased in both cell types in a dose-dependent manner; however, the increase was more pronounced in ATF3 KO cells than in wild-type MEFs. IL-6 levels in the control group were 4.1±0.2 pg/mL and 4.9±0.0 pg/mL in wild-type and ATF3 KO cells, respectively, and increased to 166.4±3.8 pg/mL and 328.8±24.3 pg/mL, respectively, upon treatment for 24 h with 8 µM doxorubicin.

### ATF3 Silencing using siRNA Reduced the Cytotoxicity of Doxorubicin in HK-2 Cells

mRNA expression levels of ATF3 decreased 54.2% in the HK-2 cells treated with ATF3 siRNA compared to scrambled siRNA control after 48 h (Figure 10A). ATF3 silencing using siRNA increased the expression of IL-6 induced by doxorubicin, indicating that IL-6 was negatively regulated by ATF3 (Figure 10B). Furthermore, ATF3 knockdown reduced the expression of cytochrome C (Figure 10C). Fluorescence-activated cell sorting (FACS) analysis showed that ATF3 silencing decreased the fraction of cells in the sub G1 phase (cell death) from 23.9% to 18.8%, indicating the reduced cytotoxicity (17.7%) by doxorubicin (Figures 10D and 10E).

## Discussion

Nephrotoxicity is one of the inherent adverse effects of certain anti-cancer drugs and anthracycline antibiotics. However, the exact mechanisms of the side effects caused by these drugs are complex and still somewhat unclear. Doxorubicin is known to interact with DNA by intercalation, causing inhibition of macromolecular biosynthesis, and to inhibit the role of the enzyme topoisomerase II during transcription and replication. In this study, doxorubicin induced the accumulation of cells in the sub G1 and G2/M phases in the human kidney proximal tubule cell line HK-2.

Although the exact mechanism of doxorubicin-induced nephrotoxicity remains unknown, it is believed to be mediated through free radical formation, iron-dependent oxidative damage of biological macromolecules, and membrane lipid peroxidation [29]. NO is a free radical gas that acts both as a cytoprotective or cytotoxic agent; the pathophysiological response is determined by concentration, time of exposure, and cell type. While a small amount of NO has a beneficial role as a messenger and host defense molecule, excessive or deficient NO production can result in a pathological state. Doxorubicin induces NO synthesis in rat cardiac cells and evokes a dose-dependent increase in both NO synthase activity in the cells and nitrite levels in the culture supernatant [30]. Doxorubicin-induced apoptosis is associated with increased transcription of endothelial NO synthase [31]. Further, TNF-α, a member of a group of cytokines that stimulate the acute inflammatory reaction, is able to induce apoptotic cell death and to inhibit tumorigenesis and viral replication [32]–[34]. In this study, the secretion of NO and TNF-α increased in a dose-dependent manner in HK-2 cells, followed by cytotoxicity.

The stress response, characterized by transduction pathways and gene transcription that serve both positive and negative aspects of cell survival, is intimately involved in the outcome of ischemic and nephrotoxic damage. The cell cycle and its regulation are key components of the life and death of stressed cells throughout the kidney. Upregulation of the ERK pathway and downregulation of JNK protect renal cells from oxidant injury [35]. In this study, doxorubicin induced the accumulation of cells in the sub G1 and G2/M phases at all doses tested, and the expression of phospho-38 and JNK did not change, whereas the expression of ERK was upregulated in HK-2 cells. To evaluate the effects of doxorubicin on ATF3 activation by ERK signaling, we used a pharmacological inhibitor of ERK, U0126 (Figures 2B and C). Inhibition of ERK reduced the expression of ATF3 and cytochrome C. The number of cells in the sub G1 phase (cell death) were also significantly decreased in the presence of the ERK inhibitor, indicating that doxorubicin could induce cytotoxicity in HK-2 cells via ERK signaling.

In addition, the expression of the ATF3 protein increased in a dose- and time-dependent manner with doxorubicin treatment in HK-2 cells. ATF3 is a member of the ATF/cyclic AMP response element-binding family of transcription factors. It is an adaptive-response gene that is induced by a wide variety of stress-causing agents, including hypoxia, metabolic stress, and DNA damage; ATF3 mRNA and protein levels are not detectable under basal conditions in most cells [23], [36], [37]. Furthermore, ATF3 has been reported to affect cell death and cell cycle progression, 2 processes that regulate the growth of cancer cells. A previous study has shown that ATF3-deficient MEFs were more efficient in transitioning from G1 to S phase, compared to wild-type MEFs [36]. In this study, the viability of ATF3 KO cells was lower than that of wild-type, together with a relative accumulation in the S phase, and the protein expression of cytochrome C was stronger in KO cells than in wild-type MEFs.

ATF3 has also been shown to negatively regulate the transcription of pro-inflammatory cytokines and has important roles in the suppression of inflammatory responses to infection and allergy. For example, a substantial increase was observed in the mRNA levels of IL-6, IL-12b, inducible nitric oxide synthase (iNOS), and TNF-α in bone marrow-derived macrophages from ATF3 KO mice stimulated with LPS, and these mice died within 24 h of exposure, compared to wild-type mice, which survived until at least 36 h post-injection [38]. The production of IL-12, p40, and IL-6 was found to be higher in the ATF3 KO primary macrophages stimulated with LPS, and mRNA expression of CCL4 was higher in the peritoneal macrophages from ATF3 KO mice compared to those from the wild-type mice [38]–[40]. ATF3 inhibits IL-6 transcription by altering chromatin structure, thereby restricting access to the transcription factors [38]. In this study, IL-6 secretion clearly increased in ATF3 KO cells compared to the wild-type cells.

It has been reported that ATF3 affects cell death and cell cycle progression. However, it is not clear whether it is a negative or positive regulator. For example, ATF3 promoted apoptosis and inhibited the transition from G1 to S phase in UV-irradiated mouse fibroblasts, whereas it inhibited doxorubicin-induced apoptosis in cardiac myocytes [14], [36]. In this study, cell cycle arrest in the S and G2/M phases was more pronounced in ATF3 KO cells than in wild-type cells. Furthermore, ATF3 may play another critical function in host defense by regulating the delicate balance between proliferative and apoptotic signals that contribute to the development of cancer.

Based on these results, we suggest that doxorubicin induces cytotoxicity through an ERK-dependent pathway, and that ATF3 plays a pivotal role as a transcriptional regulator in this process.

## References

[1] O'Donnell MP, Michels L, Kasiske B, Raij L, Keane WF (1985) Adriamycin-induced chronic proteinuria: a structural and functional study. J Lab Clin Med 106: 62–67. 4009023

[2] Milner LS, Wei SH, Houser MT (1991) Amelioration of glomerular injury in doxorubicin hydrochloride nephrosis by dimethylthiourea. J Lab Clin Med 118: 427–434. 1658168

[3] Kojima S, Icho T, Hayashi M, Kajiwara Y, Kitabatake K, et al (1993) Inhibitory effect of 5,6,7,8-tetrahydroneopterin on adriamycin-induced cardiotoxicity. J Pharmacol Exp Ther 266: 1699–1704. 8371168

[4] Mimnaugh EG (1986) Potentiation by reduced glutathione of adriamycin-stimulated lipid peroxidation in kidney microsomes. Biochem Pharmacol 35: 4337–4339. 2878665 10.1016/0006-2952(86)90714-8

[5] Xu MF, Tang PL, Qian ZM, Ashraf M (2001) Effects by doxorubicin on the myocardium are mediated by oxygen free radicals. Life Sci 68: 889–901. 11213359 10.1016/s0024-3205(00)00990-5

[6] Chularojmontri L, Wattanapitayakul SK, Herunsalee A, Charuchongkolwongse S, Niumsakul S, et al (2005) Antioxidative and cardioprotective effects of Phyllanthus urinaria L. on doxorubicin-induced cardiotoxicity. Biol Pharm Bull 28: 1165–1171. 15997091 10.1248/bpb.28.1165

[7] Spallarossa P, Altieri P, Garibaldi S, Ghigliotti G, Barisione C, et al (2006) Matrix metalloproteinase-2 and −9 are induced differently by doxorubicin in H9c2 cells: The role of MAP kinases and NAD(P)H oxidase. Cardiovasc Res 69: 736–745. 16213474 10.1016/j.cardiores.2005.08.009

[8] Billingham ME, Mason JW, Bristow MR, Daniels JR (1978) Anthracycline cardiomyopathy monitored by morphologic changes. Cancer Treat Rep 62: 865–872. 667860

[9] Doroshow JH (1986) Prevention of doxorubicin-induced killing of MCF-7 human breast cancer cells by oxygen radical scavengers and iron chelating agents. Biochem Biophys Res Commun 135: 330–335. 3954778 10.1016/0006-291x(86)90981-2

[10] Dorr RT (1996) Cytoprotective agents for anthracyclines. Semin Oncol 23: 23–34. 8783663

[11] Shan YX, Liu TJ, Su HF, Samsamshariat A, Mestril R, et al (2003) Hsp10 and Hsp60 modulate Bcl-2 family and mitochondria apoptosis signaling induced by doxorubicin in cardiac muscle cells. J Mol Cell Cardiol 35: 1135–1143. 12967636 10.1016/s0022-2828(03)00229-3

[12] Fadillioglu E, Oztas E, Erdogan H, Yagmurca M, Sogut S, et al (2004) Protective effects of caffeic acid phenethyl ester on doxorubicin-induced cardiotoxicity in rats. J Appl Toxicol 24: 47–52. 14745846 10.1002/jat.945

[13] Deman A, Ceyssens B, Pauwels M, Zhang J, Houte KV, et al (2001) Altered antioxidant defence in a mouse adriamycin model of glomerulosclerosis. Nephrol Dial Transplant 16: 147–150. 10.1093/ndt/16.1.147 11209009

[14] Nobori K, Ito H, Tamamori-Adachi M, Adachi S, Ono Y, et al (2002) ATF3 inhibits doxorubicin-induced apoptosis in cardiac myocytes: a novel cardioprotective role of ATF3. J Mol Cell Cardiol 34: 1387–1397. 12392999 10.1006/jmcc.2002.2091

[15] Arany I, Megyesi JK, Kaneto H, Price PM, Safirstein RL (2004) Cisplatin-induced cell death is EGFR/src/ERK signaling dependent in mouse proximal tubule cells. Am J Physiol Renal Physiol 287: F543–549. 15149969 10.1152/ajprenal.00112.2004

[16] Brantley-Finley C, Lyle CS, Du L, Goodwin ME, Hall T, et al (2003) The JNK, ERK and p53 pathways play distinct roles in apoptosis mediated by the antitumor agents vinblastine, doxorubicin, and etoposide. Biochem Pharmacol 66: 459–469. 12907245 10.1016/s0006-2952(03)00255-7

[17] Chang WT, Li J, Haung HH, Liu H, Han M, et al (2011) Baicalein protects against doxorubicin-induced cardiotoxicity by attenuation of mitochondrial oxidant injury and JNK activation. J Cell Biochem 112: 2873–2881. 21618589 10.1002/jcb.23201 PMC3178681

[18] Navarro R, Martinez R, Busnadiego I, Ruiz-Larrea MB, Ruiz-Sanz JI (2006) Doxorubicin-induced MAPK activation in hepatocyte cultures is independent of oxidant damage. Ann N Y Acad Sci 1090: 408–418. 17384285 10.1196/annals.1378.044

[19] Bishopric NH, Andreka P, Slepak T, Webster KA (2001) Molecular mechanisms of apoptosis in the cardiac myocyte. Curr Opin Pharmacol 1: 141–150. 11714088 10.1016/s1471-4892(01)00032-7

[20] Das J, Ghosh J, Manna P, Sil PC (2011) Taurine protects rat testes against doxorubicin-induced oxidative stress as well as p53, Fas and caspase 12-mediated apoptosis. Amino Acids. 10.1007/s00726-011-0904-4 21476075

[21] Dunkern TR, Wedemeyer I, Baumgartner M, Fritz G, Kaina B (2003) Resistance of p53 knockout cells to doxorubicin is related to reduced formation of DNA strand breaks rather than impaired apoptotic signaling. DNA Repair (Amst) 2: 49–60. 12509267 10.1016/s1568-7864(02)00185-4

[22] Huigsloot M, Tijdens IB, Mulder GJ, van de Water B (2002) Differential regulation of doxorubicin-induced mitochondrial dysfunction and apoptosis by Bcl-2 in mammary adenocarcinoma (MTLn3) cells. J Biol Chem 277: 35869–35879. 12107157 10.1074/jbc.M200378200

[23] St Germain C, Niknejad N, Ma L, Garbuio K, Hai T, et al (2010) Cisplatin induces cytotoxicity through the mitogen-activated protein kinase pathways and activating transcription factor 3. Neoplasia 12: 527–538. 20651982 10.1593/neo.92048 PMC2907579

[24] Nawa T, Nawa MT, Cai Y, Zhang C, Uchimura I, et al (2000) Repression of TNF-alpha-induced E-selectin expression by PPAR activators: involvement of transcriptional repressor LRF-1/ATF3. Biochem Biophys Res Commun 275: 406–411. 10964678 10.1006/bbrc.2000.3332

[25] Cai Y, Zhang C, Nawa T, Aso T, Tanaka M, et al (2000) Homocysteine-responsive ATF3 gene expression in human vascular endothelial cells: activation of c-Jun NH(2)-terminal kinase and promoter response element. Blood 96: 2140–2148. 10979959

[26] Kim EY, Shin HY, Kim JY, Kim DG, Choi YM, et al (2010) ATF3 plays a key role in Kdo2-lipid A-induced TLR4-dependent gene expression via NF-kappaB activation. PLoS One 5: e14181. 21152039 10.1371/journal.pone.0014181 PMC2996292

[27] Park EJ, Kim H, Kim Y, Yi J, Choi K, et al (2010) Carbon fullerenes (C60s) can induce inflammatory responses in the lung of mice. Toxicol Appl Pharmacol 244: 226–233. 20064541 10.1016/j.taap.2009.12.036

[28] Park EJ, Cho WS, Jeong J, Yi J, Choi K, et al (2009) Pro-inflammatory and potential allergic responses resulting from B cell activation in mice treated with multi-walled carbon nanotubes by intratracheal instillation. Toxicology 259: 113–121. 19428951 10.1016/j.tox.2009.02.009

[29] Pritsos CA, Ma J (2000) Basal and drug-induced antioxidant enzyme activities correlate with age-dependent doxorubicin oxidative toxicity. Chem Biol Interact 127: 1–11. 10903415 10.1016/s0009-2797(00)00159-9

[30] Aldieri E, Bergandi L, Riganti C, Costamagna C, Bosia A, et al (2002) Doxorubicin induces an increase of nitric oxide synthesis in rat cardiac cells that is inhibited by iron supplementation. Toxicol Appl Pharmacol 185: 85–90. 12490132 10.1006/taap.2002.9527

[31] Kalivendi SV, Kotamraju S, Zhao H, Joseph J, Kalyanaraman B (2001) Doxorubicin-induced apoptosis is associated with increased transcription of endothelial nitric-oxide synthase. Effect of antiapoptotic antioxidants and calcium. J Biol Chem 276: 47266–47276. 11579094 10.1074/jbc.M106829200

[32] Locksley RM, Killeen N, Lenardo MJ (2001) The TNF and TNF receptor superfamilies: integrating mammalian biology. Cell 104: 487–501. 11239407 10.1016/s0092-8674(01)00237-9

[33] Aggarwal BB (2003) Signalling pathways of the TNF superfamily: a double-edged sword. Nat Rev Immunol 3: 745–756. 12949498 10.1038/nri1184

[34] Gaur U, Aggarwal BB (2003) Regulation of proliferation, survival and apoptosis by members of the TNF superfamily. Biochem Pharmacol 66: 1403–1408. 14555214 10.1016/s0006-2952(03)00490-8

[35] Safirstein RL (2004) Acute renal failure: from renal physiology to the renal transcriptome. Kidney Int Suppl: S62–66. 10.1111/j.1523-1755.2004.09110.x 15461706

[36] Lu D, Wolfgang CD, Hai T (2006) Activating transcription factor 3, a stress-inducible gene, suppresses Ras-stimulated tumorigenesis. J Biol Chem 281: 10473–10481. 16469745 10.1074/jbc.M509278200

[37] Lu D, Chen J, Hai T (2007) The regulation of ATF3 gene expression by mitogen-activated protein kinases. Biochem J 401: 559–567. 17014422 10.1042/BJ20061081 PMC1820813

[38] Gilchrist M, Thorsson V, Li B, Rust AG, Korb M, et al (2006) Systems biology approaches identify ATF3 as a negative regulator of Toll-like receptor 4. Nature 441: 173–178. 16688168 10.1038/nature04768

[39] Khuu CH, Barrozo RM, Hai T, Weinstein SL (2007) Activating transcription factor 3 (ATF3) represses the expression of CCL4 in murine macrophages. Mol Immunol 44: 1598–1605. 16982098 10.1016/j.molimm.2006.08.006

[40] Whitmore MM, Iparraguirre A, Kubelka L, Weninger W, Hai T, et al (2007) Negative regulation of TLR-signaling pathways by activating transcription factor-3. J Immunol 179: 3622–3630. 17785797 10.4049/jimmunol.179.6.3622

